# A randomised double-blind clinical trial of two yellow fever vaccines prepared with substrains 17DD and 17D-213/77 in children nine-23 months old

**DOI:** 10.1590/0074-02760150176

**Published:** 2015-09

**Authors:** Collaborative Group for Studies of Yellow Fever Vaccine

**Affiliations:** Fundação Oswaldo Cruz, Instituto de Tecnologia em Imunobiológicos, Assessoria Clínica, Rio de Janeiro, RJ, Brasil

**Keywords:** yellow fever vaccine, randomised clinical trial, immunogenicity

## Abstract

This randomised, double-blind, multicentre study with children nine-23 months old evaluated the immunogenicity of yellow fever (YF) vaccines prepared with substrains 17DD and 17D-213/77. YF antibodies were tittered before and 30 or more days after vaccination. Seropositivity and seroconversion were analysed according to the maternal serological status and the collaborating centre. A total of 1,966 children were randomised in the municipalities of the states of Mato Grosso do Sul, Minas Gerais and São Paulo and blood samples were collected from 1,714 mothers. Seropositivity was observed in 78.6% of mothers and 8.9% of children before vaccination. After vaccination, seropositivity rates of 81.9% and 83.2%, seroconversion rates of 84.8% and 85.8% and rates of a four-fold increase over the pre-vaccination titre of 77.6% and 81.8% were observed in the 17D-213/77 and 17DD subgroups, respectively. There was no association with maternal immunity. Among children aged 12 months or older, the seroconversion rates of 69% were associated with concomitant vaccination against measles, mumps and rubella. The data were not conclusive regarding the interference of maternal immunity in the immune response to the YF vaccine, but they suggest interference from other vaccines. The *failures* in seroconversion after vaccination support the recommendation of a booster dose in children within 10 years of the first dose.

Yellow fever (YF) is an acute viral disease transmitted by mosquitoes in tropical and subtropical areas in South America and sub-Saharan Africa, with mortality rates ranging from 20-50% for the severe forms of the disease (Vasconcelos 2003). YF is one of the target diseases of the International Health Regulations that is subject to international notification with the requirement or recommendation of vaccination for travellers (WHO 2005). The vaccines available in recent decades are live attenuated viral vaccines prepared with the 17D strain, with different substrains used by manufacturers (Monath et al. 2013). The seroconversion rate of the vaccine is greater than 95% (Camacho et al. 2004), but a serological correlate of protection has not been established in humans. The best evidence of vaccine efficacy is based on epidemiological studies showing a dramatic reduction in the incidence of infection after vaccine introduction (WHO 2013a).

Immunisation of residents of endemic or threatened areas, along with tourists and visitors of these areas, is a recommendation of the National Immunisation Programme (PNI) of the Brazilian Ministry of Health (MS/SVS 2014) and the World Health Organization (WHO 2013a).

YF vaccines have demonstrated strong immunogenicity in adults, but studies have demonstrated lower seroconversion rates in infants (Grupo Colaborativo do Programa Nacional de Imunizações para o Estudo da Soroconversão pela Vacina contra Febre Amarela 2003, Gotuzzo et al. 2013, Monath et al. 2013). Moreover, lower antibody titres observed in younger children compared to adults can result in a shorter duration of immunity. The accumulation of susceptible or partially protected individuals may undermine the control of the endemic. Therefore, the recommendation of the WHO and Pan American Health Organization that the YF vaccine is valid for life (WHO 2013a) may not be suitable for endemic regions with routine immunisation of infants.

This study aimed to estimate and compare the seroconversion rates and antibody titres against YF 30 or more days after vaccination with substrains 17DD and WHO 17D-213/77 in children aged nine-23 months. There are few studies comparing the immunogenicity of vaccines from different substrains and those studies included only adults (Camacho et al. 2004). Because the 17DD substrain is only used by the Brazilian producer, a vaccine with a 17D substrain was included in the study, considering the implications for vaccine production and immunisation programmes. Comparative studies demonstrating the performance of the 17DD substrain are important for international organisations that authorise the producers of YF vaccines (WHO) or organisations that obtain vaccine for use in other countries (United Nations Children’s Fund).

The immunogenicity of the vaccines against YF was also compared in subgroups of children whose mothers were either seropositive (and did not receive the vaccine after delivery) or seronegative for YF and in subgroups of children who were vaccinated against measles, rubella and mumps up to 15 or more days apart from the YF vaccine. The frequency of adverse events in the first 30 days after vaccination was estimated and compared between vaccines.

## SUBJECTS, MATERIALS AND METHODS

*General study design* - The methods were described in detail in a previous study (Collaborative Group for Studies with Yellow Fever 2007) and they will be briefly presented here. This Phase IV randomised, double-blind, multicentre clinical trial aimed to compare the immunogenicity and reactogenicity of two different vaccines against YF prepared with substrains 17DD (licensed commercial product) and WHO 17D-203/77 (experimental product manufactured from the WHO seed-lot), both produced by Institute of Immunobiology (Bio-Manguinhos), Oswaldo Cruz Foundation (Fiocruz). The study was conducted in the Brazilian states of Minas Gerais (MG), Mato Grosso do Sul (MS) and São Paulo (SP) where YF vaccinations were scheduled in the basic immunisation calendar of the Brazilian Ministry of Health. Health care facilities were selected based on the large volume of immunisations performed and the availability of staff to participate in the study. The Coordinating Study Centre was headquartered in the Clinical Advisory of the Bio-Manguinhos.

Children whose serological tests showed no antibodies against YF 30 days after vaccination (seronegative) were revaccinated. Mothers of children aged less than one year who agreed to participate in the study were invited to provide blood samples for measuring antibodies against YF.

The study was funded by the Brazilian PNI (covenant SVS/MS 514918), Fiocruz and the National Council for Scientific and Technological Development - (479663/2004-1 and 307868/2003-6). The study was conducted according to the principles of the Declaration of Helsinki and Good Clinical Practice (OPS 2005). The protocol (CAAE - 0038.1.011.000-03) and informed consent form were approved by the Fiocruz Research Ethical Committee (CEP-Fiocruz document of approval 236A/03) and State Health Departments [CEP-SES-DF document of approval 069/2005; CEP-UFMS (Federal University of Mato Grosso do Sul)] letter of approval 738/2006). The protocol was registered at ClinicalTrials.gov (trial registration ISRCTN72367932).

*Eligibility criteria* - Mothers and caretakers of children eligible for YF vaccination were invited to participate in the study when they spontaneously attended the selected public health care facilities. Children aged nine-23 months without a history of YF vaccination available for blood sample collection 30 days after vaccination and with an informed consent form signed by parents or guardians were included in the study. Enrolment considered the contraindications to the vaccine: severe malnutrition, transient or permanent immunosuppression induced by diseases, immunosuppressive drugs, radiotherapy (topical or inhaled corticosteroids for less than two weeks did not lead to exclusion from the study, but these factors were recorded in the questionnaire), therapy with immunoglobulin or other blood products, administration of experimental vaccine 60 days before the study or an administration scheduled within 60 days after the study, history of hypersensitivity to chicken eggs (and their derivatives) or gelatin, chronic or acute severe diseases (mothers’ report or report available in the health care service) and fever (axillary temperature of 37.5ºC or higher) on the day of vaccination.

The following amendments were incorporated into the protocol: the maximum age for participation was changed from 10 years to 23 months because the number of unvaccinated children above two years was minimal; the minimum interval for post-vaccination serological testing was reduced from 45 to 30 days based on the available kinetics data for post-vaccination antibodies.

*Vaccination* - The YF vaccines produced by Bio-Manguinhos were: (i) the 17DD substrain that was being used across the country and (ii) a vaccine manufactured from the WHO17D-213/77 seed-lot, successfully used in a randomised, placebo-controlled study (Camacho et al. 2004). The distribution, handling and administration of vaccines followed the recommendations of the manufacturer (Bio-Manguinhos) and the PNI (MS/FNS/PNI 2001). Vaccines were prepared from attenuated virus grown in specific pathogen-free chicken embryos according to the WHO standards (WHO 1998). The 17DD vaccine that is routinely used in Brazil was produced from secondary seed-lot 102 (passage # 287) of the 17DD substrain. The 17D-213/77 substrain vaccine was produced from the WHO seed-lot in an experimental batch to be used only in this study. The vaccines were produced with good manufacturing practices (GMP) and have the following composition per dose (0.5 mL): YF virus (> 1,000 LD_50_), sucrose (0.8 mg), sodium glutamate (4.05 mg), sorbitol (8.5 mg), hydrolysed bovine gelatin (5.0 mg), erythromycin (3.0 mcg) and kanamycin (10.0 mcg). The diluent (water for injection) was the same used in routine YF vaccination: lot 04UDFA161Z, expiration date 04/2009. The lots and expiration dates of the vaccines used were as follows: (i) 17DD vaccine, seed-lot 993FB013Z, lot 055VFA054P, expiration date 05/2007 and (ii) 17D vaccine, WHO (17D: 213/77) seed-lot, lot 04UVFAEX34, expiration date 12/2006.

Cold chain was used for the transport and storage of vaccines and the temperature was carefully monitored at all stages of the study. In the collaborating centres and health care facilities where the vaccines were administered, the vaccines were kept in refrigerators only for vaccine storage (2-8ºC) in properly identified containers.

The vaccine application procedures, which are described in detail in the Operations Handbook of the study, included reconstituting the lyophilised vaccine (vials of 5 doses) with diluent in 2.5-mL vials immediately before application. The vaccine and diluent were kept at temperatures between 2-8ºC and were protected from light. After the application of one dose from each vial, the vials were discarded. Following the subcutaneous administration of a single dose (0.5 mL) in the deltoid region, children were observed for approximately 30 min at the health care centre with equipment and a team prepared for any acute reaction.

The vaccinators were selected according to their experience in vaccine rooms; they were trained for the specific study procedures and were supervised during the field work by nurses from the vaccination unit. Daily storage conditions (maximum and minimum refrigerator temperatures) and preparation of the vaccine for use were recorded.

*Outcomes* - The primary outcome of the study was based on the levels of antibodies against YF. The antibody titre in the serum was measured using the plaque reduction neutralisation test (PRNT), which is considered the most sensitive and specific test for YF (WHO 1998, Niedrig et al. 1999). Serum aliquots from blood samples collected immediately before and 30 days or more after vaccination were labelled with numeric codes and submitted to the PRNT in the Viral Technology Laboratory (LATEV) of Bio-Manguinhos which has extensive experience with this procedure. Seropositivity was defined as an antibody titre ≥ 2.8 log_10_mIU/mL obtained by the reference serum calibrated with the international standard (NIBSC 2011). Seroconversion was defined as (i) seropositivity after vaccination in individuals who were seronegative (titres < 2.70 mIU/mL) in the pre-vaccination serological tests and (ii) a ratio of post-vaccination/pre-vaccination titres ≥ 4 (intention-to-treat analysis). Criterion (i) above disregarded borderline titres (2.70 ≤ titre < 2.80) to avoid false negative results. These complementary forms of analysing the outcomes enabled better use of the serological data.

All vaccinated volunteers were included in the safety analysis, which evaluated events that occurred up to 30 days after vaccination. During the second visit, when post-vaccination blood was collected, the investigators asked the parents about the occurrence of signs and symptoms after vaccination. Moreover, the events that occurred during the first 10 days after vaccination were recorded on a standardised form completed by the parents. Severe adverse events (resulting in hospitalisation, death or risk of death and disability, even when temporary) were reported immediately throughout the study period.

*Randomisation and blinding* - The participants were randomised in comparison groups (vaccines from the 17D-213/77 and 17DD substrains) at an 1:1 ratio in permuted blocks of six that were generated by computer and stratified by centre (state). The vaccine vials were labelled with numbers assigned in a randomisation list. The participants received the vaccine from the vials labelled with the sequence of natural numbers as they presented for vaccination. Therefore, the participants were allocated randomly and blindly to one of the groups. Only one of five doses of vaccine from each vial was used and the study participant was identified with the number of the vial. The participant, the research team and the data analyst did not know what type of vaccine was used. Both types of vaccines and their vials looked identical. The statistician who generated and maintained the randomisation list was not involved in the data analysis. Opening the codes and breaking the blind before the conclusion of the study could be allowed by the steering committee if deemed necessary to manage severe adverse events.

*Sample size and data analysis* - The number of participants in each group was calculated based on the following parameters: 80% statistical power, 95% significance level, two-tailed test, seroconversion rate of 90% in one group (p1), relevant minimum difference in seroconversion between the groups (p1-p2) of 5% and 20% correction for losses (Fleiss 1986). With 650 children in each group, differences of up to 0.3 log_10_ in the mean titres of antibodies against YF between groups could be detected with 80% power. For adverse events with frequencies of up to 5%, the size of the proposed sample had 82% power to detect a 5% difference between the groups. For more rare events, the sample would have the power to detect larger differences.

The vaccines were designated by codes in the database, so the analyst did not know the type of vaccine administered. The analysis of the adverse events considered the entire cohort, including participants that did not adhere to the protocol (“intention-to-treat” analysis in the full cohort). For the immunogenicity analysis, the full cohort consisted of all randomised subjects who had post-vaccination serological tests. A secondary analysis of the subset that adhered to the protocol excluded individuals who received other vaccines, hyperimmune sera or drugs categorised in the exclusion and elimination criteria, seropositive individuals on pre-vaccination serology, individuals outside the age range of the study, individuals who had blood collected for serology outside the time window specified in the protocol and individuals who had clinical complications that could affect the immune response to vaccination or who had broken the blind for any reason.

The rates of seroconversion (ratio of post-vaccination/pre-vaccination titres ≥ 4), the geometric mean titres (GMTs) and the frequency of adverse events were estimated at 95% confidence intervals (CIs). To determine the GMTs, individuals with antibody titres below the serological test threshold received a value corresponding to half the threshold value.

Reverse cumulative distribution curves and histograms of the logarithms of antibody titres were built to compare the immunogenicity of the two vaccines over the entire range of titre values. The seroconversion rate was compared between the vaccinated groups and the statistical significance was assessed using the chi-square test. The mean logarithms (base 10) of the post-vaccination antibody titres were compared using Student’s *t* test.

The effect of the maternal serological status on children’s immune status was analysed by the seropositivity before vaccination and the proportion of children who did not seroconvert after vaccination, stratified by type of vaccine. The analyses of immunogenicity and reactogenicity were stratified by collaborating centre.

The frequency, time of the beginning and duration of adverse events were tabulated by type of event and group. Adverse events were analysed according to the following categories: well-known reactions to vaccine, events whose relationship to the vaccine was not known and events considered not associated with the vaccine. Regardless of the known associations, the event frequencies were compared between the groups per category of time of appearance, i.e., distinguishing the early and late events. The data were entered into an application specifically prepared for this study that generated databases formatted for analysis using SPSS software v.16.0 (SPSS, Inc, USA).

## RESULTS

A total of 1,966 children were recruited between October 2005-December 2006 in four collaborating centres that received one of the two types of vaccine against YF used in the study. The number of mothers approached is not available. The collaborating centres were the municipalities of Ribeirão das Neves and Contagem, in the metropolitan region of Belo Horizonte (n = 521), Juiz de Fora, Lima Duarte and Matias Barbosa, in MG (n = 819), Campo Grande, MS (n = 374) and Ribeirão Preto, SP (n = 252). There were no incidents, adverse events or medical care that resulted in opening the codes (breaking the blind).

Of the 1,966 volunteers who received one of the study vaccines, 14 children did not have pre-vaccination serological test results and 55 children did not have post-vaccination serological test results. The losses made up 3.2% of the participants in the 17D-213/77 vaccine group and 2.4% of those in the 17DD vaccine group and resulted from difficulties in venipuncture or missing the post-vaccination blood collection. Blood samples could not be collected from the mothers of 252 children (12.8%) because they either did not want to participate or they were not the ones responsible for the children at the time of the vaccination.

The distributions of the relevant variables were similar between the two groups before vaccination (Table I). The seropositivity in children before vaccination was substantial (Table I), ranging from 12.8-5.6% between the collaborating centres and from 8.9-9.9% in the age subgroups of eight-nine months and 12 months and older, respectively (data not shown). The majority of the mothers were seropositive for YF at rates that varied substantially among the collaborating centres (88.6-71.9%).

**TABLE I:** Characteristics of volunteers before vaccination according to the type of vaccine

	Vaccine		Total (n = 1,966)
	17D-213/77 (n = 981)	17DD (n = 985)	
Gender (% male)	50.6	51.1	50.8
Age [(months) median (max; min)]	9 (8; 22)	9 (8; 21)	9 (8; 22)
Birth weight [(g) mean (standard deviation)]	3,129 (516)	3,143 (500)	3,136 (508)
Current weight [(g) mean (SD)]	9,077 (1290)	9,148 (3259)	9,112 (2477)
Seropositivity^*a*^ PRE-vaccine [n (%)]	92 (9.4)	84 (8.5)	176 (8.9)
Maternal seropositivity^*a*^ [n (%)] (n = 1,714)	676 (79.5)	672 (77.8)	1,348 (78.6)

*a*: antibody titre ≥ 2.80 mIU/mL; SD: standard deviation.

**Fig. 1:**
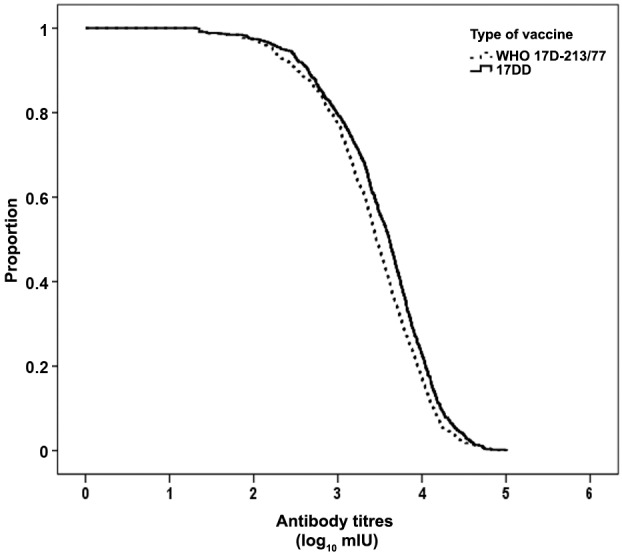
: reverse cumulative distribution of the post-vaccination antibody titres (log10 mIU/mL of serum) against yellow fever according to the type of vaccine (p = 0.0001; log-rank test).

The intensity of the immune response was slightly higher after vaccination with the 17DD substrain than with the WHO 17D-213/77 substrain, as shown in the distribution of antibody titres (Fig. 1). This pattern was similar in all four centres (data not shown). The GMTs (and 95% CI) were 3,223 mIU/mL (2,935-3,540) after the 17DD vaccine and 2,516 mIU/mL (2,291-2,763) after the 17D-213/77 vaccine. However, the magnitude of the differences between the two types of vaccine was small and not statistically significant in any of the outcomes considered in the study (Table II): proportion of post-vaccination seropositivity considering the full cohort, seroconversion by four-fold increase over pre-vaccination titres and seroconversion of seronegative to seropositive.

**TABLE II:** Number and proportion of children according to the outcome after yellow fever vaccination and the type of vaccine

	Vaccine		p
	17D-213/77 n/N (%)	17DD n/N (%)	
Seropositive individuals (≥ 2.80 mIU/mL) (full cohort)	803/981 (81.9)	820/985 (83.2)	0.184
Seroconversion among seronegative individuals before vaccination^*a*^	725/855 (84.8)	715/852 (85.8)	0.597
Ratio of post-vaccination/ pre-vaccination titres^*b*^ ≥ 4	761/981 (77.6)	806/985 (81.8)	0.131

*a*: excludes pre-vaccination antibody titres ≥ 2.70 mIU/mL and missing data; *b*: includes all with results of serologic testing.

**Fig. 2:**
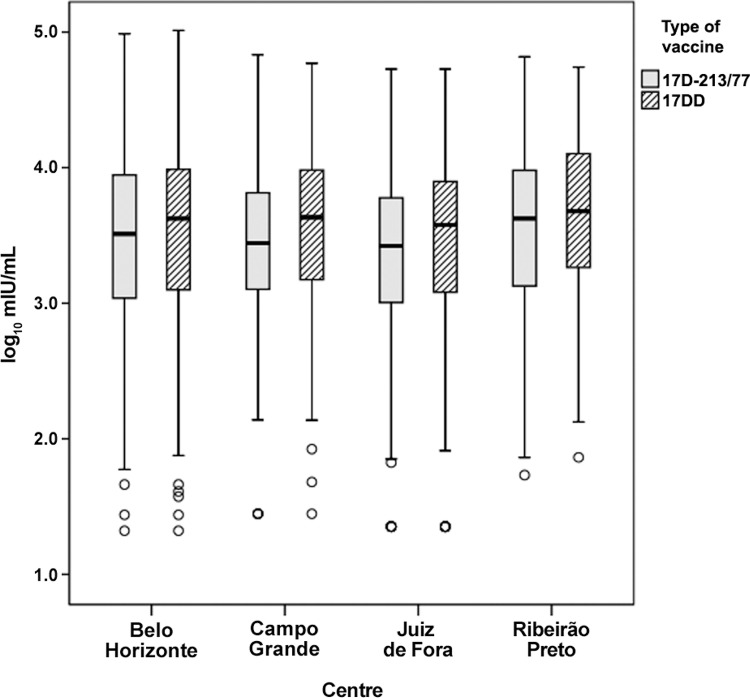
: medians and quartiles of the log10 of the antibody titres against yellow fever 30 days or more after vaccination according to the type of vaccine and collaborating centres.

In the collaborating centres, the differences between the vaccines ranged from 6.8 (Ribeirão Preto) to 0.6 (Juiz de Fora) percentage points. In the collaborating centres, the median antibody titres after vaccination ranged between 3.68 log_10_mIU/mL (17DD vaccine in Ribeirão Preto) and 3.42 log_10 _mIU/mL (17D-213/77 vaccine in Juiz de Fora) (Fig. 2). Accordingly, the proportions of post-vaccination seropositivity in those centres were 86.7% and 78.4%, respectively.

The highest proportion of seropositive children before vaccination (possibly maternal antibodies) occurred in the group of seronegative mothers, but the difference was small and not statistically significant (Table III). The proportion of seroconverted children (seronegative to seropositive) and children presenting four-fold increases over pre-vaccination titres were similar in the subgroups of maternal serological status (Table III). The proportion of seroconverted in the 17DD group was substantially higher in the subgroup of children whose mothers were seropositive, although this difference was not statistically significant.

**TABLE III:** Number and proportion of children according to serological status before and after vaccination against yellow fever, within categories of maternal serological status and the type of vaccine

Serological status of the child	Maternal serological status		p
	< 2.80 mIU/mL n/N (%)	≥ 2.80 mIU/mL n/N (%)	
Seropositive^*a*^ children before vaccination	40/363 (11)	114/1,342 (8.5)	0.137
Seroconversion^*b *^after vaccination	311/359 (86.6)	1,117/1,314 (85)	0.493
17D-213/77	144/172 (83.7)	561/655 (85.6)	0.607
17DD	167/187 (89.3)	556/659 (84.4)	0.116
Ratio of post-vaccination/pre-vaccination titres ≥ 4	295/357 (82.6)	1,082/1,313 (82.4)	0.983
17D-213/77	132/170 (77.6)	531/655 (81.1)	0.372
17DD	163/187 (87.2)	551/658 (83.7)	0.304

*a*: titres ≥ 2.80 mIU/mL; *b*: seroconversion from seronegative to seropositive.

No meaningful differences between male and female infants were disclosed in seroconversion, seropositivity, GMT and reverse cumulative curves. Differences between vaccine types in those immunisation outcomes were also very small within the strata of sex (i.e., in boys and in girls, separately). The proportion of children with a four-fold increase over pre-vaccination titres was lower among those aged 12 months or older compared to infants under 12 months of age in both vaccination groups (Table IV). Similarly, the proportions of post-vaccination seropositivity and seroconversion of individuals who were seronegative before vaccination were lower in the age group of 12 months or older than in the age group of eight-11 months (data not shown). As the combined vaccine against measles, mumps and rubella (MMR) was recommended at 12 months, a*post hoc* analysis was conducted and showed 66.2% seroconversion for YF in 68 children who received simultaneous YF and MMR vaccines and 82.6% in children who did not receive the MMR vaccine (only 8 children received the 2 vaccines on different days).

**TABLE IV:** Number and proportion of children with a ratio of post-vaccination/pre-vaccination antibody titres ≥ 4 according to the type of vaccine and age

Age (months)	Vaccine		p
	17D-213/77 n/N (%)	17DD n/N (%)	
8-9	562/691 (81.3)	618/733 (84.3)	0.155
10-11	152/191 (79.6)	148/170 (87.1)	0.080
>12	47/68 (69.1)	40/58 (69)	0.985

*Adverse events* - A total of 25.9% and 25.5% of children who received the 17D-213/77 and 17DD vaccines, respectively, presented signs or symptoms, either systemically or at the injection site, regardless of the presumed association with vaccination. Of the 505 children with some type of adverse reaction, 38.6% (17D-213/77) and 39.8% (17DD) received medical care. Fever was the most common adverse event (16.8% and 15.8% in the 17D-213/77 and 17DD vaccines, respectively). Of the children who had fever, 44.2% in the 17DD group and 40.6% in the 17D-213/77 group received medical care (not necessarily only due to fever). Vomiting was reported in 3.1% of children, with no significant difference between the two groups. Two severe events temporally associated with vaccination were reported. In the first, in Juiz de Fora, a nine-month-old child had one seizure 4 h after receiving the YF vaccine used in the study and had a second episode during his/her stay in the emergency department. Lumbar puncture was not performed and the child was discharged two days later. After three months, the child did not present abnormalities and was medicated for anaemia and epilepsy. In the second event, also in Juiz de Fora, 6 h after vaccination, a nine-month-old child presented with a 39.5ºC fever, diarrhoea, vomiting, fever, cough with hoarseness, postnasal drip, refusal of diet offered, hydrated and absence of nuchal rigidity. Complete clinical remission was observed a few days after treatment. A causality assessment based on WHO classification (WHO 2013b) indicated inconsistent causal association to immunisation in both cases.

## DISCUSSION

Immunogenicity variations among strains are known in other live attenuated viral vaccines and had been shown for YF 17D vaccine substrains in adults (Pfister et al. 2005). In the present study, differences in the intensity of the immune response to the 17D-213/77 and 17DD substrains showed no significant impact on either seropositivity or reactogenicity in children, which is in agreement with studies in adults (Camacho et al. 2004) and in 12-month-old children (Nascimento-Silva et al. 2011). The confirmation that the immunogenicity and reactogenicity of the 17DD substrain were similar to those observed in other substrains is relevant because Brazil is the only country using the 17DD substrain.

The data from the immunisation programme indicating high YF vaccination coverage in children (unpublished data) led us to change the protocol of this study, limiting the target age range to children under two years. In fact, the study group consisted predominantly of children under the age of 12 months, which constituted the majority of children spontaneously brought to health care facilities for the YF vaccination. Therefore, this study could not analyse possible differences in the immune responses between infants and children over 18 months, with the latter group being expected to be less influenced by maternal immunity and to exhibit greater maturity of the immune system.

Seropositivity for YF before vaccination (9% in this study) has been reported with a frequency of 0-19% in previous paediatric studies (Meyer Jr et al. 1964, Ruben et al. 1973, Yvonnet et al. 1986, Lhuillier et al. 1989, Mouchon et al. 1990, Soula 1991, Adu et al. 1996, Stefano et al. 1999, Osei-Kwasi et al. 2001, Belmusto-Worn et al. 2005, Nascimento-Silva et al. 2011). These studies were performed over five decades in different populations in Africa and South America, with different age groups and serological methods as well as potential differences in nutritional status, previous exposure to other flaviviruses and maternal vaccination history or natural YF infection. None of those studies presented serological data, vaccination or infection history of mothers. In general, pre-vaccination seropositivity can be explained by the transplacental transfer of maternal antibodies to the foetus and it can interfere with the immune response to vaccination in infants (Niewiesk 2014). Monitoring of children whose mothers had been vaccinated against YF during pregnancy revealed neutralising antibodies for YF in 89.4% of these children shortly after birth (Suzano et al. 2006). These neutralising antibodies were likely of maternal origin because none of the new-borns were positive for IgM and 95% of the mothers had IgG antibodies against YF. At 12 months of age, 7% of these children were seropositive and 12% presented with indeterminate serology (2.7 log_10_ mIU/mL). In the present study, the proportion of mothers who were seropositive for YF at the time of vaccination was high (79%), which is consistent with the vaccination recommendation for the areas in which they lived. However, maternal immunity was not associated with the seropositivity of children before vaccination. It must be considered that the maternal serological status at the time of vaccination of the children was a proxy of the serological status at the time of delivery. This assumption seemed reasonable because there had not been any recent immunisation campaigns or outbreaks of the disease in those areas. It was also convenient, considering that verification of the maternal immunisation history from the vaccine record is difficult in adults and does not inform about immunity by natural infection. Breastfeeding, which was not assessed in this study, can also affect the response to vaccination, either favouring (Greenberg et al. 1994) or inhibiting immunity (Pichichero 1990).

The interference of heterologous immunity in the immune response or in the antibody titration methods has been a concern in areas with circulation of other flaviviruses. Although plausible, this interference has not been confirmed in previous studies conducted in areas endemic for dengue (Belmusto-Worn et al. 2005, Martins et al. 2013, Monath et al. 2013). Data on flavivirus infections other than dengue and YF in humans are scarce in Brazil and neighbouring countries and indicate that flaviviruses do circulate. Except for rare and limited outbreaks, they have not raised public health concerns. Even if flaviviruses besides dengue and YF posed undisclosed public health problems, it is unlikely that it would be concealed for so long if its magnitude were substantial. Thus, it seems legitimate to assume that flavivirus interference has not played a major role in the serological status of children who participated in this study.

The WHO recommendation of YF vaccination from nine-12 months of age is based on safety data and risk of infection (WHO 2013a) and does not consider the differences in the immune response in this age group compared to adults. The immaturity of the immune system in the first year of life has been recognised as a limiting factor for the immunogenicity of various vaccines (Siegrist 2013) and may partially explain the results of this study that showed lower seroconversion and seropositivity rates compared to adults. The seroconversion rates observed in this study were comparable to those observed in Peru (88.5% with YF-VAX^®^vaccine) in children nine-18 months old (Belmusto-Worn 2005) and in Brasília, the Federal District (DF) (88.8% with 17DD vaccine) in children 13 months old in which the YF vaccine was administered at least 30 days after the MMR vaccine (Nascimento-Silva et al. 2011). In the latter study, children who received the MMR and YF vaccines on the same day (in separate injections) had an even lower seroconversion rate (70%) than children who received these vaccines 30 days apart. Lower seroconversion rates compared to those of adults (67.9-84.6%) were also observed in SP (Stefano et al. 1999), where nine-month-old children received YF and measles vaccines at intervals ranging from one-28 days. In Upper Volta (Meyer Jr et al. 1964), a study analysed children aged five-54 months (approximately 75% of them younger than 12 months) vaccinated with a needle-free injector (“jet injector”). A seroconversion rate of 97% was observed in the subgroup of children who received the YF vaccine alone and a rate of 85% was observed in children who received a combination of vaccines against measles, smallpox and YF in the same syringe. In Mali (Soula et al. 1991), a study reported a seroconversion rate of 92.7% in infants aged four-eight months who received a combination of vaccines against YF and measles. Additionally, a combination of measles and YF vaccines in Côte d’Ivoire (Lhuillier et al. 1989) resulted in seroconversion rates of 88% and 91% in infants younger than seven months and older than eight months, respectively. In Nigeria (Ruben et al. 1973), children aged six-24 months (34% of them aged 6-11 months) who received simultaneous measles, smallpox and YF vaccines in separate applications with a “jet injector” had a seroconversion rate of 96.6% for YF. A subgroup who received measles, smallpox, YF and diphtheria/tetanus/pertussis (DTP) vaccines had a seroconversion rate of 94.8%. In Nigeria (Adu et al. 1996), infants aged six-eight months had a seroconversion rate of 87.1% when they received a simultaneous application of the YF and measles vaccines and a rate of 93.1% with the YF vaccine alone. In these subgroups of vaccines, children aged nine-12 months presented with very similar seroconversion rates (95.7% and 96.9%, respectively). In Senegal (Yvonnet et al. 1986), children aged nine-36 months who received a simultaneous application of the hepatitis B, measles, polio and DTP vaccines in separate injections had seroconversion rates of 91.5-93.6% for YF in subgroups with different mean ages (18.3 and 26.2 months). In Ghana (Osei-Kwasi et al. 2001), the seropositivity of infants aged six and nine months was 98.6% and 98%, respectively, three months after the YF vaccination. In a multicentre observational study in children (mean age 9.3 months) in Senegal and Guiana (Michel et al. 2015), 92.9% of children vaccinated simultaneously against YF and measles and 90.7% vaccinated seven-28 days apart were seropositive.

Other studies performed in children older than 12 months of age have reported post-vaccination seropositivity rates against YF of 94% in the Central African Republic (Georges et al. 1985), 94.7% in Mali (Soula et al. 1991) and 94.6-96.6% in Peru (Vacina Arilvax^®^) (Belmusto-Worn et al. 2005).

The great diversity of scenarios, methodological approaches and other features not specified in previous studies precludes clearly distinguishing what determines the differences observed in the levels of seropositivity and antibody titres after vaccination. Relevant elements, such as the power of the vaccine, mode of administration, concomitant application of other vaccines (particularly the live attenuated viral vaccines) and the serological method used, varied widely and may have interfered in the evaluation of the immunogenicity of vaccines.

The three outcomes established in this study sought to help with the challenges in immunogenicity measurement using imperfect tests. The inaccuracy in the proportion of seropositivity results from the application of cut-off points in the titres and misclassifications in both pre and post-vaccination tests. The proportion of seroconversion after excluding seropositive individuals before vaccination was also affected by the limitations of seropositivity classification. The derivation of seroconversion from the variation of the antibody titres of each individual before and after vaccination reduces the impact of the serological test inaccuracy and highlights the capacity of the vaccine to increase the pre-existing titres. In contrast, seroconversion arbitrarily defined as a four-fold increase over pre-vaccination titres may be less sensitive in detecting weak responses.

The variations in the results obtained in the collaborative centres of this study did not affect its conclusions and can be attributed to differences in the age distribution and in the pre-vaccine immunity and maternal immunity profiles. Considerable effort was invested in the standardisation of the study procedures, thus minimising the contribution of the methodology to the differences (e.g., vaccination technique and measurement of antibody titres) in the results among the centres.

Despite the limitations on comparability with previous studies, most of the studies have reported a lower seropositivity in infants than what is generally observed in adults and older children. The eligibility criteria for routine vaccination excluded some of the most important conditions that interfere with the immune response, such as immunodeficiency and malnutrition, which therefore do not explain the lower seroconversion rates relative to adults. Infants who would have been eligible for routine vaccination may have been excluded by the conservative criteria of clinical studies. Thus, the result is a healthier-than-average study population vaccinated under controlled conditions, which may optimise the performance of the vaccine.

The reactogenicity profile of the vaccines in this study was similar to that in other studies regarding the frequency and nature of events (Belmusto-Worn et al. 2005, Nascimento-Silva et al. 2011). The systemic events were nonspecific and cannot be distinguished from the clinical problems common in this age group. Perhaps the variations in the frequencies of these complications explain part of the differences between the studies. The signs and symptoms at the injection site also occurred with expected frequencies.

The data from this study are not conclusive regarding the interference of maternal immunity on the immune response to the YF vaccine. Interference from other vaccines applied simultaneously, particularly those from live attenuated viruses, has been suggested in several studies cited above. Regardless of the mechanism, the evidence that the YF vaccination may fail to seroconvert a significant proportion of infants indicates that the recommendation to vaccinate every 10 years or that one dose of vaccine protects throughout one’s lifetime needs to be reviewed. This issue has been controversial, which prompted an Advisory Committee on Immunization Practices Work Group (Centers for Disease Control and Prevention) to conduct an additional review of the available evidence (Staples et al. 2015). As a result, the recommendation for booster doses was discontinued for most travellers but was kept for travellers who plan to spend a prolonged period in endemic areas or those travelling to highly endemic areas.

The Brazilian PNI has maintained vaccination at nine months of age and anticipated booster vaccination at four years of age based on epidemiological and programmatic criteria and published studies on the vaccine (MS/SVS/DVDT/CGPNI 2014). A second dose was not considered appropriate during the second year of life, which already has several other vaccines for diseases considered more relevant than YF for public health. Postponing to four years of age (~3 years after the 1st dose) averted the busy immunisation schedule, without adding significant risk of disease. In fact, from 1990-2009, 21 cases of YF below five years of age were reported in Brazil. From 2007-2012, seven cases have been reported in children below 15 years of age, all of them unvaccinated (Martins & Homma 2014). Future studies should investigate whether individuals will need to be vaccinated indefinitely every 10 years after the second dose of YF vaccine.

## COLLABORATIVE GROUP FOR STUDIES OF YELLOW FEVER VACCINE

*Steering committee* - Luiz Antonio Bastos Camacho (Principal Investigator), Marcos da Silva Freire, Maria da Luz Fernandes Leal, Maria de Lourdes de Sousa Maia and Reinaldo Menezes Martins (Fiocruz).

*Study site coordinators* - Helena Keico Sato (State Health Secretary, SP), Guilherme Côrtes Fernandes (Santa Casa de Misericórdia, Juiz de Fora, MG), Ivone Perez de Castro (Health Secretary, DF), José Geraldo Leite Ribeiro (State Health Secretary, MG), Jandira Campos Lemos (State Health Secretary, MG), Eugenio Oliveira Martins de Barros (Health Secretary, Campo Grande, MS) and Anna Maia Yamamoto Yoshida (LATEV).

*Collaborators* - Takumi Igushi (Fiocruz), Márcia Borges Leitão (State Health Secretary, MG), Maristela Batista (Health Secretary, Juiz de Fora, MG), Maria da Conceição Barros (Health Secretary, Campo Grande, MS), Emilia Nakamatsu (Health Secretary, Campo Grande, MS), Elisabete Paganini (State Health Secretary, SP), Marileide Nascimento (Fiocruz), Nilce da Silva (Fiocruz), Sirlene de Fátima Pereira (MS/SVS/DEVIT/CGPNI, Brazilian Ministry of Health), Ernesto Isaac Montenegro Renoiner (MS/SVS/DEVIT/CGPNI, Brazilian Ministry of Health), Marilia Ferraro Rocha [MS/SGEP/DENASUS, Manaus, state of Amazonas (AM)], Marly Almeida Galdino (MS/SGEP/DENASUS, Manaus, AM), Luiza de Marilac Meireles Barbosa (University of Brasília) and Zouraide Guerra Antunes Costa (MS/SVS/DEVIT/CGDT, Brazilian Ministry of Health).

*Roles and responsibilities of the members - *Research protocol development: LABC, HKS, MSF and MLFL; coordination and oversight of field work: LABC, MLSM, HKS, GCF, JCL, EOMB, MB, MCB, EM, EP, MN and NS; coordination and oversight of serological tests: AMYY; data analysis and interpretation, review and approval of final version of the paper: LABC, RMM, MSF, MLFL, MLSM, HKS, GCF, EOMB, JCL and SFP.

*Disclaimer* - MSF, MLFL, MLSM and RMM are staff members of Bio-Manguinhos, a technical unit responsible for manufacturing the YF vaccine; AMYY was the head of the Laboratory at Bio-Manguinhos, which performed the laboratory tests; LABC and TI are staff members of the Brazilian National School of Public Health, a technical unit of Fiocruz.
